# A Physician’s View of One Health: Challenges and Opportunities

**DOI:** 10.3390/vetsci2010023

**Published:** 2015-02-16

**Authors:** Barbara Natterson-Horowitz

**Affiliations:** Professor of Medicine, Division of Cardiology, David Geffen School of Medicine at UCLA, Los Angeles, CA 90095, USA; E-Mail: bnatterson@mednet.ucla.edu; Tel.: +1-310-825-5280

One Health is one of the most important movements and emerging concepts in health today. The convergence of the fields of human and animal medicine has the potential to generate novel scientific hypotheses, create effective new therapies and potentially transform how physicians, veterinarians and their patients understand health and disease. Despite this potential, One Health has not yet gained significant awareness or traction in human medical communities. From its inception, One Health, sometimes also called One Medicine, has been piloted primarily by leaders from the world of veterinary medicine. Although the specific term was coined perhaps 10 years ago, comparative medicine has been quietly evident on university campuses with veterinary and medical schools for decades longer. Although a few physicians have played major leadership roles in One Health, in the United States, despite over ten years of the movement’s robust growth, many have still not heard of it. Furthermore, physicians with some awareness of One Health often believe it to be primarily and exclusively about zoonotic infections and global health. The much broader scope and potential of One Health as also including comparative physiology and medicine is not being communicated effectively. Consequently, the human medical community remains largely disengaged. This is problematic because without significant engagement from physicians, nurses and other human health care professionals, the potential of One Health cannot be realized. To advance One Health it is imperative that we first understand the roots of under-engagement of the human medical community. This, in turn, can guide the development of novel and engaging opportunities for physician which demonstrate the power relevance of One Health’s comparative, collaborative and cooperative approach.

One of the most fundamental challenges to bringing individual physicians into One Health is to demonstrate that it is relevant to their daily practice of medicine. It is important to remember that the vast majority of physicians are not infectious disease specialists or public health officials. They are internists, pediatricians, obstetrician-gynecologists, cardiologists, dermatologists, and oncologists, among others. There are countless connections between the physiology and pathology of humans and animals in all of these fields. Yet, an informal survey of One Health conferences and journals over the past two years reveals content that is dominated by infectious disease, zoonoses, food safety, and environmentally-related concerns [1,2]. While these issues are tremendously important to human populations, the relative paucity of content from other comparative medical fields is a lost opportunity. Demonstrating the many intersections between animal and human health which are non-infectious is essential to counter physician under-engagement in One Health.

How can the human medical community’s awareness and engagement in One Health be strengthened? I faced this question ten years ago as I became increasingly aware and interested in One Health. At the time, I was a faculty cardiologist at the David Geffen School of Medicine at UCLA. I became fascinated by the intersection of human and veterinary medicine. Through my lecturing about One Health to groups of medical students and physicians, I found that while there was mild interest in the topic in general, interest surged when I focused the on comparative studies of the diseases they were dealing with in their human patients. I began tailoring my One Health lectures to their areas of focus and interest. Indeed, when speaking with cardiology fellows, I would discuss hypertrophic cardiomyopathy in cats, mitral regurgitation in Cavalier King Charles spaniels, and ventricular tachycardia in boxers [3,4,5]. When speaking with psychiatry residents, the discussion would focus on compulsive disorders in Doberman pinchers, feather plucking in parrots and separation anxiety in young dogs [6,7,8]. I began targeting the content of my lectures to help these physicians-in-training recognize the deep connections between the two medical fields. This tailored, specialty-specific approach led to many requests by students for clinical and research opportunities in One Health.

The increased interest that followed these lectures led to the creation of the Zoobiquity Conferences in 2011 [9]. The conferences are designed to bring together physicians and veterinarians taking care of the similar diseases in different species. The past four U.S.-based conferences cases have included sessions about breast cancer, obsessive-compulsive disorder, heart failure, melanoma, self-injury, infertility and many others. The Zoobiquity Conferences are designed to feature the benefits of the comparative method and to formulate models of interdisciplinary collaboration. For example, a veterinarian presented a case of glioblastoma multiforme in a Rhodesian ridgeback; a physician also presented a case of glioblastoma in a high school principal. They shared with one another imaging, histology, treatment protocols and outcomes through an engaging discussion about similarities in people and non-human animals. The audiences at the conferences comprised of nearly equal distributions of professionals from clinical human and veterinary medicine. The conferences also include a medical field trip in which attendees are led “on rounds” by leading academic and zoological veterinarians at local zoos and on veterinary school facilities.

Developing programs and educational opportunities tailored for physicians is crucial to the development of One Health in the coming years. Helping physicians recognize that the diseases they treat in their patients are often not uniquely human and that animal health experts have important insights to share will advance the One Health concept. Programming which exposes practicing physicians to veterinarians taking care of the same disorders in their patients is one effective way to drive this point home.

These conversations and debates provide a necessary boost to pull One Health from the periphery of the human medicine to the central position where it belongs. There is so much opportunity, yet the gap between the two professions persists. Taking stock of where the barriers are and developing programming to remove them, will do much to promote One Health across the health professions.

## References

[1] One Health Initiative “Events”. http://www.onehealthinitiative.com/events.php.

[2] One Health Initiative “Publications”. http://www.onehealthinitiative.com/publications.php.

[3] Kittleson M.D., Meurs K.M., Munro M.J., Kittleson J.A., Liu S., Pion P.D., Towbin J.A. (1999). Familial Hypertrophic Cardiomyopathy in Maine Coon Cats: An Animal Model of Human Disease. Circulation.

[4] Borgarelli M., Savarino P., Crosara S., Santilli R.A., Chiavegato D., Poggi M., Bellino C., La Rosa G., Zanatta R., Haggstrom J., Tarducci A. (2008). Survival Characteristics and Prognostic Variables of Dogs with Mitral Regurgitation Attributable to Myxomatous Valve Disease. J. Vet. Intern. Med..

[5] Basso C., Fox P.R., Meurs K.M., Towbin J.A., Spier A.W., Calabrese F., Maron B.J., Thiene G. (2004). Arrhythmogenic Right Ventricular Cardiomyopathy Causing Sudden Cardiac Death in Boxer Dogs: A New Animal Model of Human Disease. Circulation.

[6] Dodman N.H., Karlsson E.K., Moon-Fanelli A., Galdzicka M., Perloski M., Shuster L., Lindblad-Toh K., Ginns E.I. (2010). A canine chromosome 7 locus confirms compulsive disorder susceptibility. Mol. Psychiatry.

[7] van Zeeland Y.R.A., Spruit B.M., Rodenburg T.B., Riedstra B., van Hierden Y.M., Buitenhuis B., Korte S.M., Lumeij J.T. (2009). Feather damaging behaviour in parrots: A review with consideration of comparative aspects. Appl. Anim. Behav. Sci..

[8] Flannigan G., Dodman N.H. (2001). Risk factors and behaviors associated with separation anxiety in dogs. J. Am. Vet. Med. Assoc..

[9] Zoobiquity Conference Overview. http://zoobiquity.com/conference-research/conference/conference-overview/.

